# Simulation on neonatal stabilization and transport

**DOI:** 10.1186/1824-7288-41-S1-A5

**Published:** 2015-09-24

**Authors:** Armando Cuttano, Rosa T Scaramuzzo, Francesca Moscuzza, Davide Panizza, Massimiliano Ciantelli, Marco Vuerich, Emilio Sigali, Antonio Boldrini

**Affiliations:** 1 Centro di Formazione e Simulazione Neonatale “NINA”, U.O. Neonatologia - Azienda Ospedaliero Universitaria Pisana, Italy; 2 Istituto di Scienze della Vita, Scuola Superiore Sant'Anna di Pisa, Italy; 3 Università di Pisa, Italy

## 

Legal problems are more numbered even in Neonatology.

Hospital policy should establish strategies to limit systematic, environmental and human errors, including solutions to optimize organization and increase knowledge [1].Staff retraining by means of simulation could be a precious instrument.

Simulation works through mechanisms that are proper of our brain for decision-making (i.e. simulated mind) [2]. The capacity of evaluating a situation earlier than it happens and so planning possible actions is the so called anticipatory simulation [3]. Physiological basis of simulation effectiveness are deeply related to mirror neurons [4]. Simulation emphasizes the so called “deutero-learning”, i.e. the context in which (proto-)learning processes occur: at the same time people are learning simple concepts, and also learning something about the world and about how things occur [5]. In sum, simulation maximises learning through the extraction of implicit rules, and putting specific bits of basic experiences in context to generalise them.

The aim of this lecture is to focus on methods of simulation on neonatal stabilization and transport (more than on medical procedures themselves).

Simulation retraining for medical (or nurse) staff should be based on the andragogic approach: adults are conscious of their own educational needs and focus their own attention on specific interests (related to daily practice) [6]. This methodological approach needs that teachers behave as peers towards learners, with empathy and a collaborative attitude.

A precious additive methodological element is fun: the serious medical game is the approach to optimize technical memories trough emotions [5].

In our experience, materials for simulation are daily clinical devices and innovative stuff developed by a multidisciplinary collaboration [7];we discuss published guidelines (e.g. STABLE Program) and participants’ experiences.

As regards neonatal stabilization and transport, we perform: i) annual retraining sessions for all the operators in our Unit, ii) low and medium fidelity simulation sessions for nearby hospitals, iii) high fidelity sessions for colleagues working at geographically uncomfortable hospitals (i.e. island) and so needing for transport by helicopter, as a kind of “full scale CRM (Crisis Resources Management)”.

In our opinion the traditional approach to teaching is inadequate for retraining of adult professionals. We propose simulation as the method to deepen knowledge, strengthen abilities and so optimize performances. Recording sessions and analysing them to discuss behaviours is a main instruments for debriefing. Every kind of support (e.g. e-learning platforms, papery stuff, pocket memory materials, etc) is admitted and should be creatively promoted.
